# Anthropometric estimates can predict satisfaction with breast in a population of asymptomatic women

**DOI:** 10.1186/s41687-024-00814-9

**Published:** 2024-11-28

**Authors:** Giuseppe Catanuto, Valentina Di Salvatore, Concetta Fichera, Patrizia Dorangricchia, Valeria Sebri, Nicola Rocco, Gabriella Pravettoni, Francesco Caruso, Francesco Pappalardo

**Affiliations:** 1 Humanitas University, Milan, Italy; 2 Università degli Studi di Catania, Catania, Italy; 3 Associazione Sant’Antonese per la Lotta ai Tumori, Aci Sant’Antonio, Catania, Italy; 4 Applied Research Division for Cognitive and Psychological Science, IEO, European Institute of Oncology IRCCS, Milan, Italy; 5 Department of Advanced Biomedical Sciences, University of Naples “Federico II”, Via Sergio Pansini 5, 80131 Naples, Italy; 6 Department of Oncology and Hemato-Oncology, University of Milan, Milan, Italy; 7 Department of Health and Drug Sciences, University of Catania, Catania, Italy

**Keywords:** Breast surgery, Anthropometric measures, Quality of life

## Abstract

**Background:**

Several authors hypothesized that normative values of breast related quality of life in asymptomatic populations can be helpful to better understand changes induced by surgery. Breast related quality of life can be associated to breast anthropometry. This study was designed to explore this hypothesis, find relevant correlations and, using machine learning techniques, predict values of satisfaction with breast from easy body measurements.

**Methods:**

Asymptomatic women undergoing routine clinical examination for breast cancer prevention were interviewed using the BREAST_Q V1 Breast Conserving Surgery Pre-op. Descriptive statistics was performed to describe the characteristics of the population. The Pearson correlation test defined correlation between relevant anthropometric variables and scores in each domain of the BREAST_Q. Regression analysis was employed to assess variation in the “Satisfaction with breast” domain when looking at the mirror dressed or undressed. Three machine learning algorithms were tested to predict scores in the “Satisfaction with breast domain” given body mass index and nipple to sternal notch distance.

**Results:**

One-hundred and twenty-five women underwent clinical examination and assessment of anthropometry. The reply rate to the BREAST_Q ranged from 99.2 to 88% depending on the domains. The “satisfaction with breast” domain was negatively associated either to BMI [r_Pearson_ = −0.28, CI (−0.41, −0.15) *p* < 0.005] and Age [r_Pearson_ = −0.15, CI (−0.29, −6.52e-03) *p* = 0.04]. The N_SN distance was also negatively associated to this domain with the following values for the right [r_Pearson_ = −0.34, CI (−0.45, −0.21) *p* < 0.000] and left side [r_Pearson_ = −0.31, CI (−0.43, −0.17) *p* < 0.000]. Linear regression analysis was performed on questions 1 and 4 of the “Satisfaction with Breast” domain revealing a steeper decrease for women with higher BMI values looking in the mirror undressed (Adjusted R-squared BMI: Dressed − 0.03329/Undressed − 0.08186). The combination of two parameters (BMI and N_SN distance) generated the following accuracy values respectively for three machine learning algorithms: MAP (Accuracy = 0.37, 95% CI: (0.2939, 0.4485)); Naïve Bayes (Accuracy = 0.70, 95% CI: (0.6292, 0.7755); SVM (Accuracy = 0.63, 95% CI: (0.5515, 0.7061)).

**Conclusions:**

This study generates normative scores for a Mediterranean population of asymptomatic women and demonstrates relevant associations between anthropometry and breast related quality of life. Machine learning techniques may predict scores of the “satisfaction with breast” domain of the Breast_Q using body mass index and nipple to sternal notch estimates as input. However, the algorithm seems to fail in approximately one third of the sample probably because is not able to capture many aspects of personal life. Much larger sample and more qualitative research is required before establishing any direct association between body estimates and quality of life. Clinical implications are given.

## Introduction

Quality of life serves as a crucial measure in assessing the overall well-being and satisfaction of patients undergoing surgical treatment for breast cancer [1]. This encompasses various aspects such as physical, emotional, social, and functional well-being. By utilizing questionnaires both before and after surgery, healthcare professionals can comprehensively evaluate breast-related quality of life. These assessments provide valuable insights into the impact of surgery on patients’ daily functioning, psychological state, and interpersonal relationships, aiding in the development of personalized treatment plans and supportive care interventions [2–5].

Several authors hypothesized that normative values of breast related quality of life in asymptomatic populations can be helpful to better understand changes induced by surgery [6–8]. Populations with different racial and socioeconomic status may retain different scores and for this reason baselines should be ideally calculated at a local level [9–13].

Breast related quality of life can be also dependent on the ethnicity and the specific morphotype of a population [14–18].

The Breast-Q is an organ specific questionnaire designed and validated to assess quality of life in patients undergoing breast surgery for cancer treatment. Questions are subdivided in four domains each one related to specific aspects of personal satisfaction: psycho-social; physical; psycho-sexual and satisfaction with breast [19].

This study aims to define normative values of Breast_Q within a Mediterranean population of asymptomatic women and find associations with anthropometric measures.

According to the WHO definition, anthropometry is a simple, portable, universally applicable, inexpensive and non-invasive technique for assessing the size, proportions and composition of the human body [20].

In this regard, literature shows the relevant role of Machine Learning (ML). As a definition, ML is focused on developing algorithms and statistical models that improve computers’ performance on specific tasks through data analysis and pattern recognition. Instead of being explicitly programmed for each task, ML systems learn from input data and autonomously adjust their algorithms to increase the accuracy of their predictions or classifications, mimicking the human learning process. This iterative learning process allows models to adapt and optimise as they encounter new information, making them particularly effective in applications such as speech recognition, computer vision, predictive analytics, and other areas where data complexity and variability demand dynamic and flexible solutions [21].

In the present study, we used this technology to predict scores of the “satisfaction with breast” domain starting from anthropometric measures retaining the highest correlation values.

## Methods

After review and approval of the institutional board of Associazione Santantonese per la Lotta ai Tumori, (a charity organization dealing with patients’ advocacy and cancer prevention based in Aci Sant’Antonio, Catania, Italy) asymptomatic women attending clinical examination for breast cancer prevention were submitted to the pre-op Breast-Q questionnaire (BREAST_Q V.1 Breast Conserving Surgery Pre-op. Italian Translation). Upon completion a physician (GC) collected the following estimates: height (cm); weight (kg); nipple to sternal notch (N_SN) distance (cm, right and left); areola to infra-mammary fold (A_IMF) distance (cm, right and left); distance between nipples (N_N) (cm, right and left). Combining these values, we obtained three more measures: Delta N_SN; Delta A_IMF, Body mass index (BMI)

### Statistical analysis

Exploratory analysis with descriptive statistics was performed for assessment of key characteristics including the distribution of age, height, weight, and body type across the entire sample.

Linear correlation analysis was used to examine relationships between variables by calculating Pearson’s correlation coefficients. Pearson’s correlation index, often denoted as *r*, quantifies the strength and direction of a linear relationship between two continuous variables. Its values range from −1 to +1, where *r* *=* *−*1 indicates a perfect negative linear correlation (one variable decreases as the other increases), *r* *=* 0 implies no linear relationship, and *r* *=* *+*1 indicates a perfect positive linear correlation (both variables increase together in proportion).

In this study, a significance threshold of *p* *<* 0.05 was applied to determine whether the observed correlations were statistically meaningful. A scatter plot provided a two-dimensional visual representation of the relationship between BMI and “Satisfaction with Breast,” showing how data points align in a pattern that can reveal both the direction and strength of their correlation.

A square correlation matrix was generated to visually represent the strength and direction of the correlation coefficient between each pair of variables. This matrix helps identify potential linear relationships across variables by displaying correlation values, where positive or negative coefficients indicate the direction of association.

To further investigate specific relationships, particularly between BMI and satisfaction scores from the Breast_Q questionnaire, regression analysis was conducted. This analysis focused on question 1 (satisfaction when viewing oneself in the mirror when dressed) and question 4 (satisfaction when undressed), both within domain 1 of Breast_Q. Breast_Q scores were divided into five categorical classes to assess trends across satisfaction levels, and the adjusted R-squared values were reported to evaluate the proportion of variance explained by the model, accounting for the number of predictors.

Finally, p-values were adjusted to control for multiple comparisons, enhancing the statistical robustness of the findings and minimizing the risk of type I errors.

### Machine learning

A supervised classification was used to predict scores of “satisfaction with breast” based on BMI alone and in combination with N_SN distance, as these measures showed the highest correlation with this domain. Supervised classification is a machine learning approach where an algorithm is trained on labeled data (in this case, known scores of satisfaction with breast) to recognize patterns or relationships that can be used to predict outcomes for new, unseen data.

Three specific machine learning algorithms were tested in this analysis: Maximum a Posteriori Probability, Naïve Bayes, and Support Vector Machine (SVM). Each of these algorithms applies different strategies for classification. For instance, Maximum a Posteriori Probability calculates the likelihood of each outcome given the data, while Naïve Bayes uses probabilistic methods assuming feature independence, and SVM aims to find an optimal boundary between classes for accurate classification. By using these algorithms, the analysis sought to accurately predict satisfaction scores from the selected features.

The performance of each classifier was evaluated using four key metrics: *accuracy, sensitivity, specificity*, and *confidence interval. Accuracy* measures the overall correctness of the classifier, calculated as the proportion of correct predictions (both true positives and true negatives) out of all predictions. *Sensitivity*, or recall, assesses the model’s ability to correctly identify true positive cases (how often it predicts the positive class when the actual class is positive). *Specificity* measures the classifier’s ability to correctly identify true negative cases, or how often it correctly predicts the negative class when the actual class is negative. The *confidence interval* provides a range within which the true value of these metrics is expected to lie, giving a level of statistical confidence in the reliability of these estimates.

*Receiver Operating Characteristic (ROC) curves* were also used to assess classifier performance more comprehensively. An ROC curve visualizes the trade-off between sensitivity and specificity across different threshold settings for the classifier. By plotting the true positive rate (sensitivity) against the false positive rate (1 - specificity), the ROC curve illustrates how well the classifier distinguishes between classes. The *Area Under the ROC Curve (AUC)* provides a single value summarizing the classifier’s ability to separate classes; a value closer to 1 indicates high discriminatory power, while a value near 0.5 suggests that the classifier performs no better than random guessing.

Together, these metrics offer a multi-dimensional view of each model’s performance, helping to determine its practical applicability and robustness.

The models were classified as follow:

- AUC ≈ 0.5: Poor discrimination (similar to random guessing).
- AUC > 0.5: Better-than-random discrimination.
- AUC = 1.0: Perfect discrimination.

The entire analysis, from exploration to classifier design, was conducted using the Bioconductor R programming language [19].

## Results

One-hundred and twenty-five women underwent clinical examination and assessment of anthropometry. The same population was submitted to the breast q questionnaire. Not all the individuals replied to all the domains (124, 99.2% replies psychophysical domain and 110, 88% replies psychosexual domain).

The characteristics of the population are described in Tables 1 and 2.

**Table 1:** Anthropometry of the population

	Mean	Std.Dev	Min	Median	Max
Height	160.5	5.3	149	160	175
A_IMF Right	7.8	2.3	4	8	14
A_IMF Left	7.7	2.2	2	8	13
BMI	25.4	4	17.2	24.9	35.5
DELTA A_IMF	0.1	0.9	−3	0	4
DELTA N_SN	−0.1	1	−4	0	3
N_N	22.6	2.7	18	22	31
Age	60.6	8	51	59	86
N_SN Right	25.4	3.6	18	25	38
N_SN Left	25.6	3.5	19	25	37
Weight	65.6	10.5	45	65	95

**Table 2:** Breast-Q values

	Mean	Std.Dev	Min	Median	Max
Physical	48.9	7.4	14	50	60
Satisfaction with breast	51.9	12.2	23	53	100
Psychosexual	61.5	14	31	60	100
Psychosocial	59.8	18.9	0	59	100

**Fig. 1:**
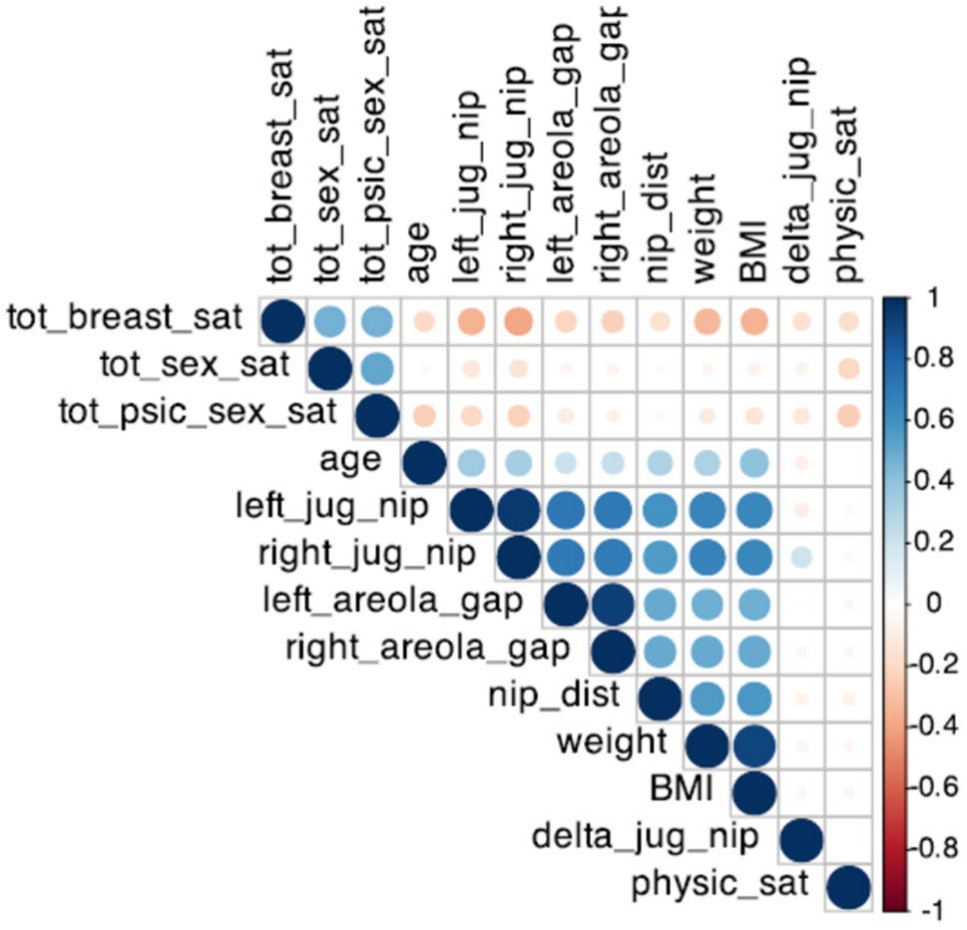
This correlation matrix displays the correlation coefficients between variables related to general satisfaction and anthropometric measurements. Each cell shows the correlation between two variables, with blue shades indicating positive correlations and red shades indicating negative correlations. Darker colors signify stronger correlations, either positive or negative, as indicated by the color bar on the right

The results of the Pearson correlation test are visualized in the correlation matrix visible in Fig. 1.

**Fig. 2:**
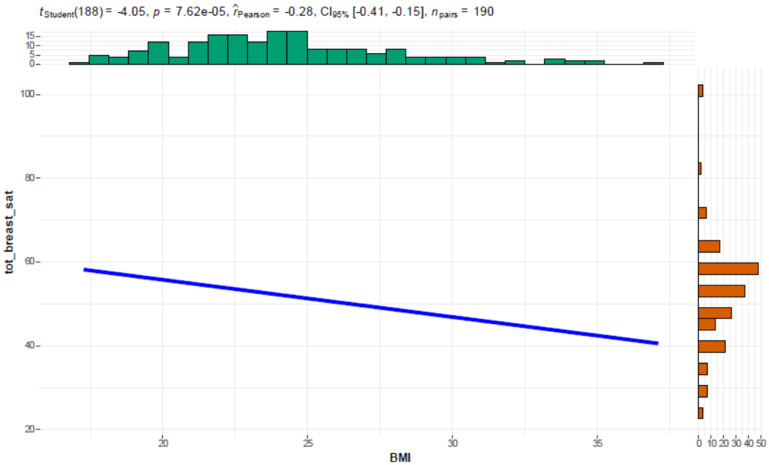
Correlogram plot showing the Pearson correlation between BMI and satisfaction with breast appearance. The data reveals a statistically significant negative correlation, suggesting that higher BMI is associated with lower satisfaction. Histograms for BMI (top) and satisfaction scores (right) provide distributions for each variable

**Fig. 3:**
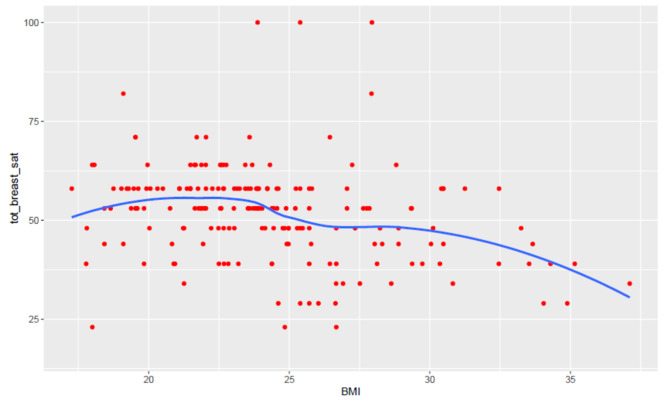
Scatter plot showing the relationship between BMI and satisfaction with breast appearance. Each red point represents an individual observation, and the blue line is a locally estimated scatterplot smoothing (LOESS) curve, which suggests a trend of decreasing breast satisfaction with increasing BMI. This visualization highlights the non-linear nature of the relationship, with satisfaction levels appearing relatively stable at lower BMI values and declining as BMI increases beyond 25

The “satisfaction with breast” domain was negatively associated either to BMI [r_Pearson_ = −0.28, CI (−0.41, −0.15) *p* < 0.005] and Age [r_Pearson_ = −0.15, CI (−0.29, −6.52e-03) *p* = 0.04] (Figs. 2, 3).

The N_SN distance was also negatively associated to this domain with the following values for the right [r_Pearson_ = −0.34, CI (−0.45, −0.21) *p* < 0.000] and left side [r_Pearson_ = −0.31, CI (−0.43, −0.17) *p* < 0.000].

Correlation was performed also within the set of anthropometric estimates. The sternal notch-nipple distance was strongly positively associated to the areola to IMF distance [r_Pearson_ = 0.69, CI (0.61, 0.76) *p* < 0.000], as well as the distance between nipples [r_Pearson_ = 0.57, CI (0.47, 0.66) *p* < 0.000]. All the distances increased with the increase of BMI.

**Fig. 4:**
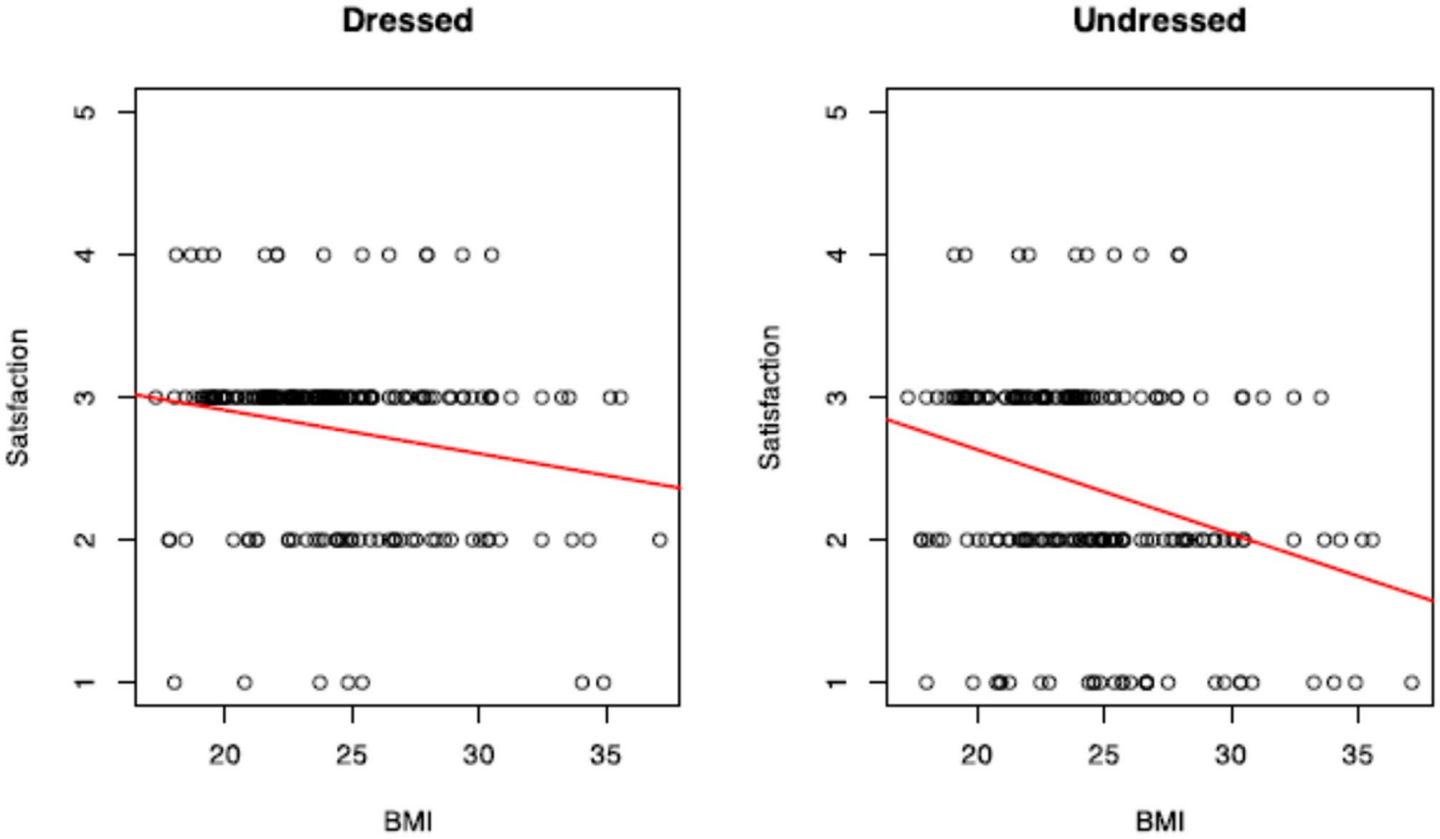
Linear regression analysis of breast satisfaction vs. BMI when looking at oneself dressed (left) and undressed (right). Each plot includes individual satisfaction scores on a 1–5 scale, with the red regression line indicating a negative trend. The results suggest that as BMI increases, satisfaction with breast appearance decreases, both when dressed and undressed. However, the slope appears more pronounced in the undressed condition, potentially indicating greater dissatisfaction with breast appearance at higher BMIs when viewing oneself without clothing

**Fig. 5:**
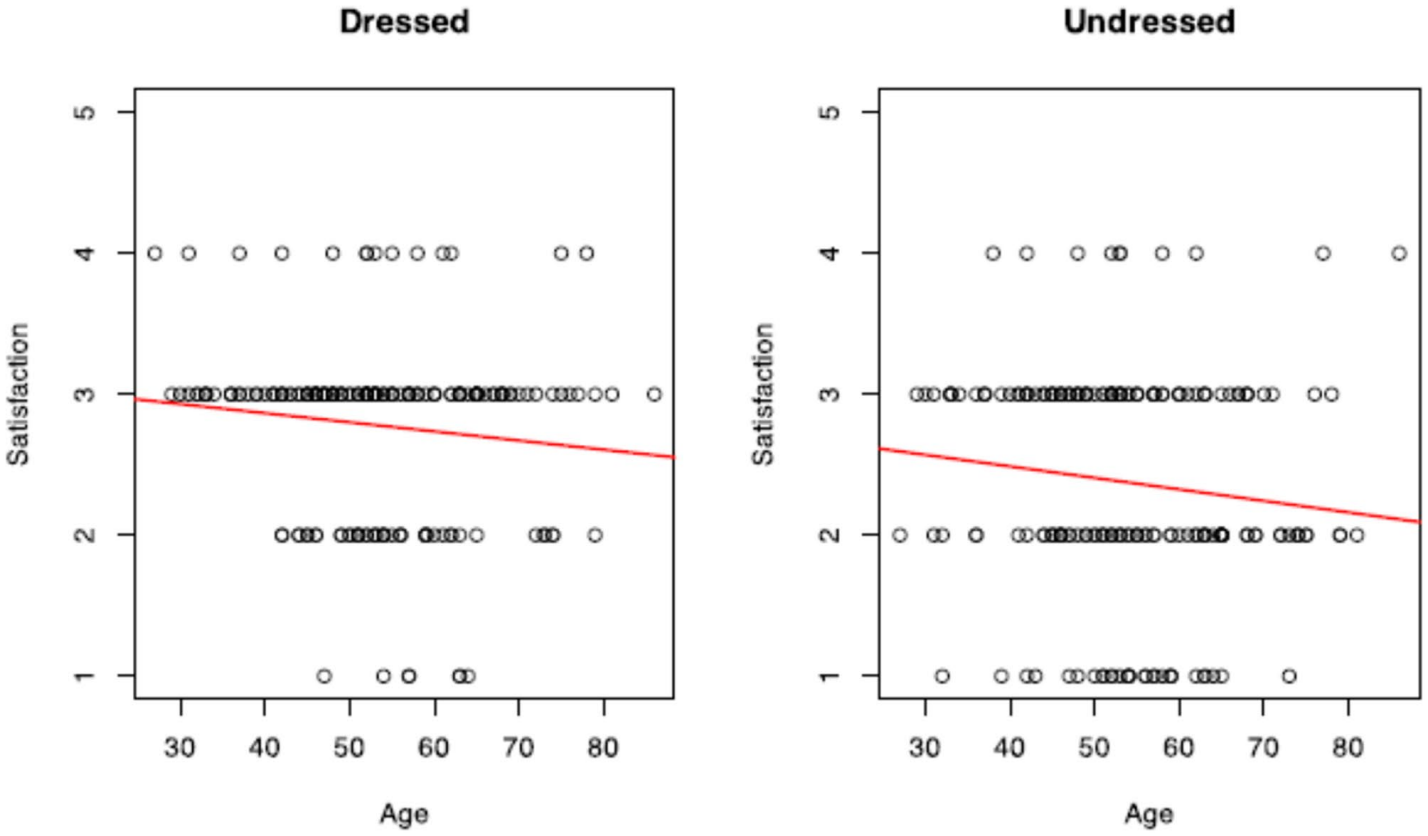
Linear regression analysis of breast satisfaction vs. age when viewing oneself dressed (left) and undressed (right). Each plot displays individual satisfaction scores on a 1–5 scale, with a red regression line showing a negative trend. The results indicate that as age increases, satisfaction with breast appearance tends to decrease, both when dressed and undressed. However, the slope is steeper in the undressed condition, suggesting that dissatisfaction with breast appearance may be more pronounced at higher ages when viewing oneself without clothing

Linear regression analysis was performed on questions 1 and 4 of the “Satisfaction with Breast” domain. Scores decreased with the increase of BMI and Age (Figs. 4, 5). The decrease was steeper for women with higher BMI values looking in the mirror undressed (Adjusted R-squared BMI: Dressed − 0.03329/Undressed − 0.08186).

**Fig. 6:**
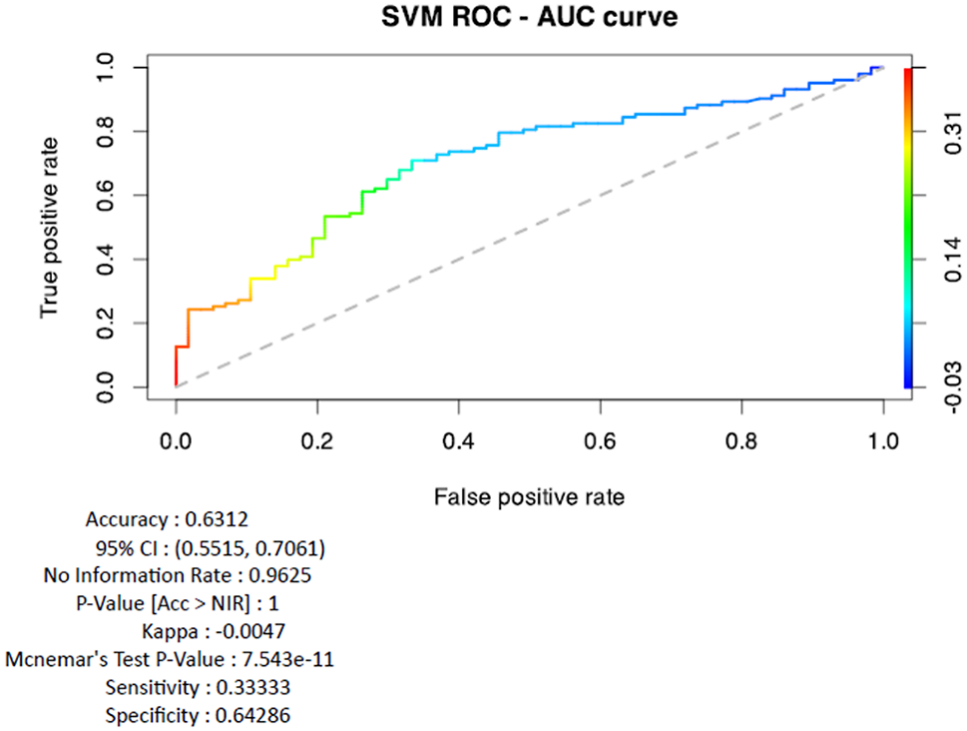
Support Vector Machine (SVM) AUC Curve and Performance Metrics. The area under the curve (AUC) value, calculated as AUC_ROCR_SVM, is ≈0.71, indicating a good predictive performance

Finally, predictions of scores of “Satisfaction with Breast” given a BMI value were performed using maximum a posteriori probability (MAP), Naïve Bayes and Support Vector Machine (SVM). Accuracy values for MAP were 0.66 95% CI: (0.54, 0.77); for Naïve Bayes 0.69 95% CI: (0.5747, 0.7976); for SVM 0.70 95%CI: (0.58, 0.8095) (Fig. 6).

**Fig. 7:**
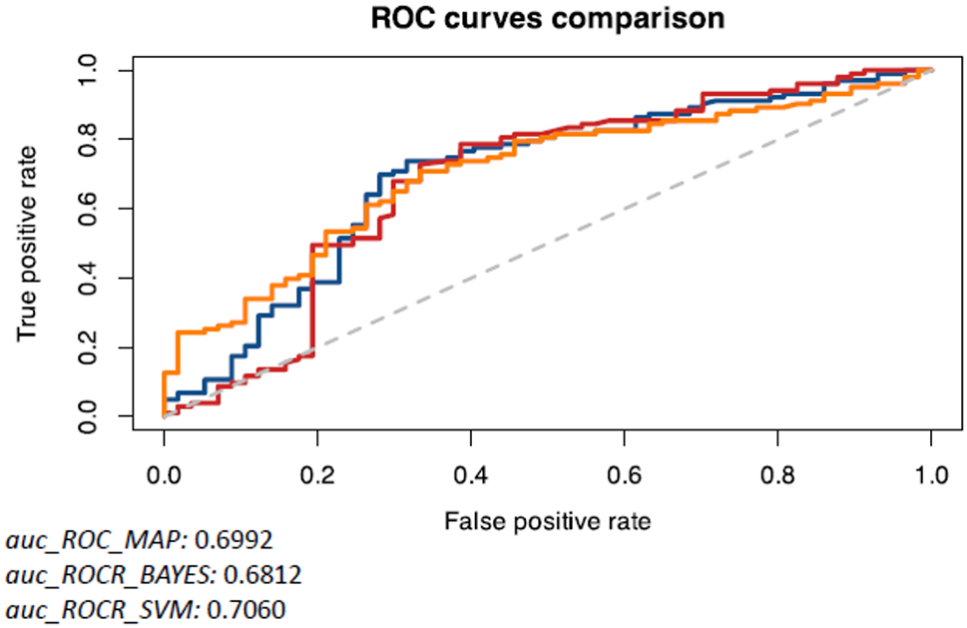
Comparison of AUC curves and performance metrics of the different algorithm. The area under the curve (AUC) value is higher in the SVM case, indicating this approach has the best predictive performance

ROC Curves and AUC values in comparison between different algorithms are displayed in Fig. 7.

**Fig. 8:**
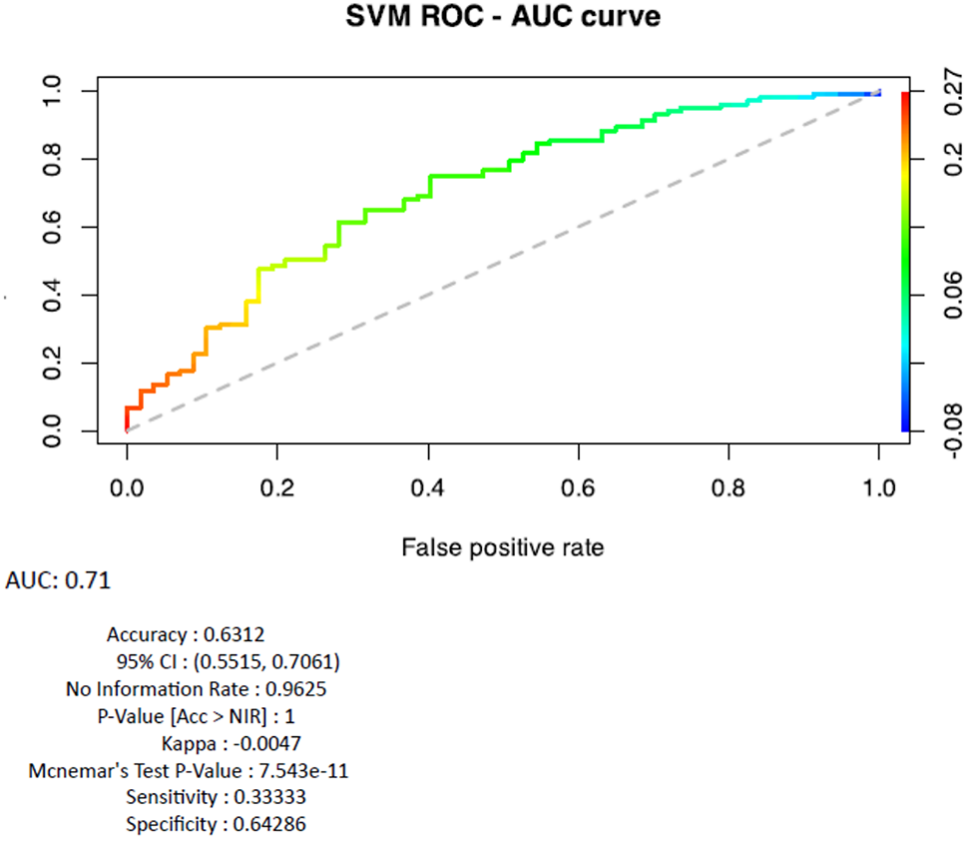
Support Vector Machine (SVM) AUC Curve and Performance Metrics calculated after the combination of two parameters (BMI and N_SN distance). The area under the curve (AUC) value, calculated as AUC_ROCR_SVM, is ≈0.63, indicating a good predictive performance

The combination of two parameters (BMI and N_SN distance) generated the following accuracy values respectively for MAP (Accuracy = 0.37, 95% CI: (0.2939, 0.4485)); Naïve Bayes (Accuracy = 0.70, 95% CI: (0.6292, 0.7755); SVM (Accuracy = 0.63, 95% CI: (0.5515, 0.7061)) (Fig. 8).

**Fig. 9:**
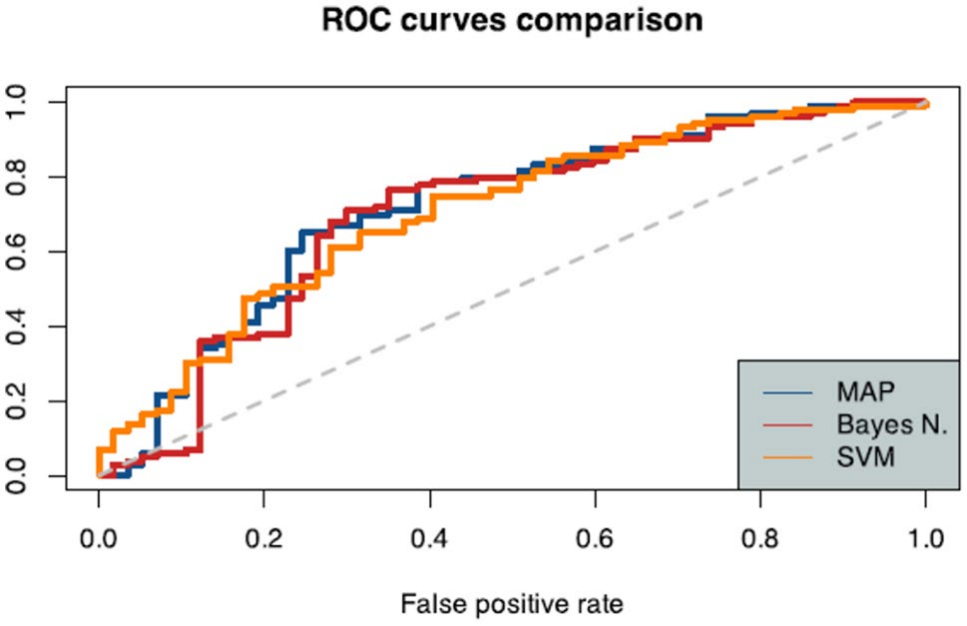
Comparison of AUC Curves and Performance Metrics of the different algorithm calculated after the combination of two parameters (BMI and N_SN distance)

The AUC values and the ROC Curves comparison is displayed in Fig. 9.

## Discussion

### The psychological burden of anthropometry

The impact of anthropometric measures on patient satisfaction, particularly in the context of surgical interventions such as breast cancer treatment, is indeed a crucial aspect to consider in healthcare. Studies have highlighted how changes resulting from surgeries can influence patients’ perceptions of their body image and overall satisfaction. Overall, body image has a strong impact on women’s psychological well-being. It belongs to bodily self, which is defined as an integrated system based on sensory-motor information [22, 23]. Factors such as weight gain and scarring may contribute to decreased satisfaction among women undergoing such procedures [24]. Moreover, Braude and colleagues underscore the importance of acknowledging patients’ potential regret following surgical interventions [25]. This regret can stem from various sources, including dissatisfaction with the outcome or emotional distress resulting from the decision to undergo surgery. Understanding these emotional issues is essential for promoting patients’ well-being and long-term satisfaction with their treatment choices. Additionally, the role of social context, as elucidated by O’Connell and colleagues, further complicates the landscape of patient satisfaction [26]. Women may internalize societal beauty standards and compare themselves to others, leading to heightened aesthetic ideals and potentially unrealistic expectations regarding the outcomes of breast cancer surgery. These high expectations can exert significant emotional pressure on patients and may ultimately influence their satisfaction with the treatment process. It is in line with the self-discrepancy theory by Higgins (1987) that demonstrated high level of emotional issues in women who perceive a huge gap between the perception of who they are and who they would like to be, which is strongly influenced also by society and its standard of beauty [27]. Effective management of patients’ expectations and emotions, as well as fostering a supportive environment, are crucial for enhancing satisfaction and promoting overall well-being among breast cancer patients undergoing oncological interventions [28].

### Anthropometry, normative values and breast related quality of life

This study aims to define normative values of Breast_Q within a Mediterranean population of asymptomatic women and find associations with anthropometric measures.

Three algorithms of ML to predict scores of the “satisfaction with breast” domain starting from anthropometric measures retaining the highest correlation values.

This study identifies breast related anthropometric characteristics of a population of asymptomatic women living in the South Mediterranean area. The median BMI value reported for this cohort is 25.5 which is comparable to what was reported for western countries [29].

The median nipple to sternal notch distance is 25 cm (either for the right and the left breast), this value is slightly higher with respect to what was reported by Mokkapati for the Indian population (23.99 cm) [30] but considerably higher compared to Chinese women [31].

The Pearson correlation test revealed a strong positive association between the sternal notch distance and the other distances, leading to the conclusion that this estimate can be representative of all the others. Similar conclusions are reported for body mass index. This work does not investigate breast volume, whose evaluation is rather complex and time consuming (requiring digital imaging or surface scanning). However, other reports had associated volume to N_SN distance and body mass index [32]. According to this study, the N_SN distance and body mass index are two easily quantifiable measures providing a relevant amount of information that eventually may be used to replace volume or other more complex estimations.

A very large proportion of interviewed women replied to the Breast-Q questionnaire except for questions related to sexual domain. Overall, this study reports low scores in satisfaction with breast concurrently with other previous data from a population of women affected by breast cancer and candidate to surgery [33].

Values of the Breast-Q for each domain in other normative studies are visible in Table 3 [7, 8, 33–35]. Some heterogeneity is revealed across different nations and this is likely to reflect different social environments, ethnicity and body anthropometry.

**Table 3:** Comparison of baseline values among other population and pre-post op values from the same author

	Sat_Breast	Psychosocial	Sexual	Physical
Italian [28]	53	59	60	50
Swedish [29]	57	66	48	84
US [30]	58	71	56	93
Dutch [7]	68	72	58	80
Australian [8]	50	55	79	42

This work theorizes that breast and body anthropometry contribute to determinate satisfaction with breast and breast related quality of life to some extent.

In the past, Jepsen et al. reported on a similar population of 146 women who answered to the Breast-Q reconstruction pre-op the questionnaire [36]. In comparison to this cohort the mean age was lower years, and the mean body mass index (BMI) was comparable (25 kg/m2). The mean score for satisfaction with breast was 57 on a 0–100 scale. This series showed that women with high BMI values seemed less satisfied with their breasts and physical and sexual well-being.

These findings are also confirmatory of a previous Australian cohort in which overweight women who completed the pre-op breast reconstruction model obtained lower scores in all the investigated domains [8].

Sadok et al in the Dutch cross sectional population survey reported that higher body mass index is negatively associated to breast satisfaction assessed using the Breast_Q pre-operative breast reconstruction module in a multivariate linear regression analysis [7].

The effects of BMI are evident even in the US “Army of women” study that confirmed that a body mass index of 30 kg/m2 or greater was associated to lower scores. Greater cup size, younger age and annual income were also determinants of reported breast related quality of life [6].

Age can be considered a further indicator of scores in quality of life. In this report the Pearson Correlation test demonstrated a weakly (tough significant) negative associationwith breast and physical satisfaction. These results are in an apparent contradiction with those reported by Sadok in the Dutch cohort, who indicated age as a protective factor towards this domain [7].

Linear regression analysis in this cohort confirmed that satisfaction with breast is dependent on body mass index and declines more quickly with increasing BMI when undressed. Similar conclusions are associated to age. Other two recent studies are concurrent with these results. Jepsen and Butt reported that a large proportion of the population was very or somewhat dissatisfied with their appearance when looking at themselves unclothed in the mirror [34, 36].

Differently from previously reported studies this work was designed to make predictions of breast related quality of life starting from breast anthropometry estimates. Considering BMI and N_SN distance the most relevant anthropometric measures according to the Pearson Correlation test, three ML algorithms were tested to predict scores of “satisfaction with breast”. The highest AUC value was obtained using the SVM algorithm (*auc_ROCR* *=* 0.7060) using BMI alone, while combining the two parameters (BMI + N_SN distance) the best performing algorithm was the MAP with an increase of AUC values to AUC = 0.72.

In other words, given one or two simple measures it is possible to anticipate breast related satisfaction precisely without administering the questionnaire in the large majority of the population.

These results are not expected to replace the interviews, but can be used in routine clinical practice to perform a quick estimation of patients’ satisfaction with breast. Considering that most of the times questionnaires are time consuming, not all patients or are willing to reply, and that normally not more than two/three interviews are performed in the follow-up, we can use anthropometric distances for a rough monitoring and, in case of changes call for a standard assessment with questionnaires.

One of the limitations of this study is that it fails to explore socio-economic details, including income, marital status, job or physical activities. All these aspects would have provided relevant information on the characteristics of the population and possibly explain low values in comparison to other normative studies.

Another limitation relies on the weak linear correlation reported for most of the studied combinations (i.e. BMI vs. Satisfaction with breast). A graphic scatter plot (Fig. 3) let us hypothesizes that a non-linear association is present and that satisfaction with breast is maximized in specific intervals outside of which a decline is expected.

## Conclusions

This study generates normative values of anthropometry and breast related quality of life for a local population of Mediterranean women. The nipple to sternal notch distance is strongly correlated with BMI and the other common anthropometric estimates on the breast. BMI and nipple to sternal notch distance are correlated to satisfaction with breast. Breast satisfaction decreases when undressed when BMI increases according to regression analysis.

ML algorithms can predict satisfaction with breast starting from BMI and Nipple N_SN distance with relatively high accuracy. These can be used for quick monitoring during routine clinical examination for follow-up.

Despite the AUC value being acceptable the ML algorithms fail in approximately 30% of the cases. This population has to be explored to understand what variables can compromise satisfaction despite optimal BMI and anthropometry. The present study suggests that algorithm does not capture some aspects of personal life as a limitation. Thus, it would be relevant to explore this topic in future studies. Additionally, further research should test anthropometry in the setting of patients who are candidate for breast surgery aiming to anticipate post-operative outcomes in quality of life starting from baseline anthropometry. Third, a larger sample size and qualitative research would be essential to define any direct association between body estimates and quality of life. Lastly, future studies would implement Artificial Intelligence and ML to better explore this field of interest, starting from their ability to value patients’ health [37].

## Data Availability

Datasets are available in a data repository and will be available on reasonable request
